# Obesity in young age is a risk factor for preeclampsia: a facility based case-control study, northwest Ethiopia

**DOI:** 10.1186/s12884-016-1029-2

**Published:** 2016-08-19

**Authors:** Mulualem Endeshaw, Fantu Abebe, Solomon Worku, Lalem Menber, Muluken Assress, Muluken Assefa

**Affiliations:** 1 Pre-service Education of Health Professionals, International Non-Governmental Organization, P.O. Box, 1566, Bahir Dar, Ethiopia; 2 Rift Valley University, Bahir Dar Campus, Bahir Dar, Ethiopia; 3 Medical Education & In-service Training of Health professionals, ICAP, Addis Ababa, Ethiopia; 4 Pre-service Education of Health Professionals, Non- Governmental Organization, Bahir Dar, Ethiopia; 5 Consortium of Christian Relief & Development Associations (CCRDA), Addis Ababa, Ethiopia; 6 Bahir Dar Health Science College, Bahir Dar, Ethiopia

**Keywords:** Preeclampsia, MUAC, Obesity, Compliance

## Abstract

**Background:**

Preeclampsia is one of the most commonly encountered hypertensive disorders of pregnancy. For many years, obesity has been suggested to play a role in preeclampsia. However, the hypotheses have been diverse and often revealed inconsistent results. This study has aimed to estimate the effect of obesity and dietary habits on preeclampsia in Bahir Dar City, north-western Ethiopia.

**Methods:**

A facility-based unmatched case-control study was conducted on 453 (151 cases and 302 controls) pregnant women, attending antenatal care or skilled delivery at Bahir Dar City. Data were collected through face to face interviews and measurements of mid-upper-arm circumference (MUAC) at the time of the interviews. Data were cleaned and entered into IBM SPSS version 20 and later analyzed using STATA version 12. Univariate and multivariate logistic regression analyses were employed to estimate the effect of independent variables on preeclampsia. Stratified analysis was conducted to check for presence of confounding and/or effect modification between covariates.

**Result:**

The odds of preeclampsia were higher among obese (MUAC ≥25 cm) women than their leaner counterparts (AOR = 3.33, 95 % CI: 1.87, 5.79). Obesity was also found to have a similar magnitude of risk for late onset preeclampsia (AOR = 3.63, 95 % CI: 1.89, 6.97). When stratified by age, the effect of obesity on overall and late onset preeclampsia was significant among young (age < 35 years) women (COR = 1.81, 95 % CI: 1.11, 2.99) and (COR = 2.09, 95 % CI: 1.16, 3.86), respectively. As the age groups became more homogenous through adjusted stratification, obesity showed a particularly significant effect in women age ≤24 and 25–29 years; (AOR = 2.31, 95 % CI: 1.06, 5.12) and (AOR = 3.66, 95 % CI: 1.37, 10.87) respectively. Similarly, the effect of obesity on late onset preeclampsia was evident among younger women age ≤24 and 25–29 years; (AOR = 3.16, 95 % CI: 1.21, 8.24) and (AOR = 1.98, 95 % CI: 1.16, 3.40) respectively. However, obesity has no significant effect on early onset of preeclampsia (AOR = 1.98, 95 % CI: 0.79, 4.94). On the other hand, compliance to folate supplementation during pregnancy and fruit consumption were associated with reduced risk of preeclampsia.

**Conclusion:**

Obesity in young age was found to be a risk factor for preeclampsia while compliance to folate supplement and adequate fruit consumption were found to be protective against preeclampsia. Promoting healthy life style, including body weight control, consumption of fruits and vegetables, and folate supplementation should be promoted to reduce the risk of preeclampsia.

## Background

Preeclampsia is defined as hypertension accompanied by proteinuria first detected after 20 weeks of gestation with or without generalized edema [1, 2]. It has remained a significant public health threat in both developed and developing countries, and contributes to maternal and perinatal morbidity and mortality globally [3, 4]. Preeclampsia has shown an increasing trend in Ethiopia with an estimated prevalence of 8.4 %, and ranks third among the top four causes of maternal mortality in the country [5–7].

Preeclampsia is characterized by metabolic disturbances similar to those found in cardiovascular diseases and type 2 diabetes mellitus (T2DM), including endothelial dysfunction, inflammation, oxidative stress, insulin resistance and dyslipidemia [8]. A multisystem nature of the syndrome with involvement of almost all organs, activation of coagulation and increased sensitivity to pressor agents have led to extensive research and understanding of the disorder in recent years [9, 10]. However, definite causes of preeclampsia have not yet been discovered.

Although the etiology of preeclampsia remains largely unclear, evidence suggests that several factors, including, but not limited to, age, body size and maternal diet play a role in the etiology of preeclampsia [8, 11]. There is compelling evidence that obesity increases the risk of preeclampsia about 3-folds, and is the leading risk factor in developed countries [12].

On the other hand, a healthy diet characterized by high intake of calcium, vegetables, fruits and vegetable oils was associated with reduced risk of preeclampsia in women [13–15]. Observational studies revealed that dietary components and qualities associated with macronutrients, micronutrients, dietary fiber, alcohol, caffeine, individual foods as well as overall food patterns have links with occurrence of preeclampsia [16–18]. Trials aimed at preeclampsia prevention, however, yielded diverse and often inconsistent results across studies [12, 19, 20].

Therefore, this study has aimed to explore the effect of obesity and dietary factors on early onset, late onset and overall preeclampsia in order to better understand the risk and protective factors of preeclampsia in the region.

## Methods

### Study design and period

A facility-based unmatched case- control study was conducted on pregnant women, attending antenatal care or skilled delivery in Bahir Dar City, Ethiopia, from June to September, 2014.

### Study area

Bahir Dar is the capital city of Amhara region; the second largest and populous region in the country located approximately 565 km northwest of the capital city, Addis Ababa.

The city has one public referral hospital, two private hospitals, 6 public health centers, and about 12 higher and medium private clinics. These facilities serve approximately 5 million people in the city and the neighboring districts. All of these facilities provide ANC and skilled delivery services. The study involved only public health facilities (a referral hospital and 6 health centers) because the characteristics of women visiting these facilities were similar and the higher proportions (98 %) of the cases were identified from them. Currently, utilization of Antenatal Care (ANC) and skilled delivery services in the region is 43 and 10.2 % respectively. Only 1/5^th^ of women who had ANC visits attend skilled delivery service [7]. The characteristics of women who attend ANC or skilled delivery service are similar with no variation in the type of health facility (hospital or health center) they visit to receive either of the services.

### Study population

Pregnant women who began attending Antenatal Care (ANC) or those who were seeking skilled delivery service during the study period were enrolled in the study. Except in case of the outcome of interest, cases and controls had similar background characteristics. The proportion of ANC attendance was equal for the cases and controls (96 % vs 95.4 %). However, complete data on the number of ANC visits in reference to controls were not obtained. There was no difference in background information between participants and non-participants.

A case refers to pregnant women who were diagnosed to have any form of preeclampsia/eclampsia during ANC follow up or delivery by a clinician (doctor, midwife or trained nurse). Preeclamptic women were identified as cases if they experience blood pressure of 140/90 mmHg or higher at least twice 4 h apart after 20 weeks of pregnancy, as well as urine laboratory result revealing protein excretion [21].

Controls are normotensive women who already delivered during the same time as cases. As soon as a case was identified, two controls were enrolled by random sampling method from the same health facility. To avoid misclassification of potential cases as controls, a 48-h post-partum waiting time was required.

Sample size was determined using EPI info stat calculator for two populations, taking 85 % power of the test, 95 % confidence level and a case to control ratio of 1: 2. The sample size was determined to detect an odds ratio of 2.3. This estimation was taken from a previous study that identified advanced age as a risk factor for preeclampsia [11]. Based on the assumption, the total sample size required for cases and controls, including a 10 % non-response was 151 and 302 respectively.

### Sampling technique and procedure

Proportional allocation of sample size was made to the participating institutions. Pregnant women who were attending ANC or delivery service during the study period and fulfilled the inclusion criteria were recruited until the allotted sample was reached. Survivor sampling method was employed to enroll the study participants. Cases were enrolled until the required sample size was obtained. For each case, two controls (women who already delivered) were selected by using a simple random sampling technique from the same health facility on the same day.

### Study variables

The presence of preeclampsia/eclampsia during pregnancy or delivery was the outcome variable, whereas maternal socio-demographic characteristics (age, marital status, education, residence, monthly income in Birr (1$ = 20 Birr) and maternal occupation), dietary habits (intake of fruit, green vegetables, meat and animal product, and folate) prior to and/or during pregnancy, nutritional status (Mid-Upper Arm Circumference (MUAC) and Hemoglobin value (Hgb) measured in the first trimester) and maternal lifestyle patterns (physical exercise, alcohol intake and coffee consumption) were the independent variables.

Based on Gestational Age (GA), preeclampsia was classified as early onset (<34 completed weeks gestation), late onset (≥34 completed weeks gestation) and overall preeclampsia [11].

### Context

Ethiopia is one of the poorest countries in the world where approximately 30 % of its population live below the poverty line (income per capita < $1.25). Eighty four percent of its people live in rural areas, mostly with rainfall dependent subsistent farming as a means of survival [22].

The staple diet for most (98 %) of the study population is Enjera (a pancake-like thin and enormous bread baked from Teff-a local cereal, wheat or maize flour) and a stew/sauce prepared from a cooked mix of milled legumes, onions and oil, taken on an average of two times per day. Despite being advised by care providers, regular intake of green vegetables, fruit and optimum amount of animal products is unaffordable to most of the population, including pregnant women despite being advised by care providers to do so.

Iron and folic acid supplement in the form of folate is universally given to all pregnant women during ANC follow up. Self-reported compliance to folate supplement varies between 70 and 86 % in the region. In our study, compliance to folate intake was determined if a woman has been taking folate tablets daily as per the WHO recommendation [23].

Intake of fruits, vegetables and animal products was considered adequate if a woman has been taking fruit, vegetables and one of the animal products at least 3 times per week 6 months prior to the detection of the pregnancy.

### Measurement of nutritional status

BMI and MUAC are often used to classify pregnant women as underweight, normal, overweight or obese by measuring nutritional status and body size The use of BMI at about 10 weeks of gestation to assess nutritional status in adults, including pregnant women has been recommended in developing countries [24]. However, pregnant women in the study area did not book early antenatal care; therefore, their actual weights at 10 weeks of gestation were not available. Thus, MUAC was used to assess body size, as it was considered to be stable during pregnancy [25]. Based on literatures classification [25, 26], we classified MUAC value as underweight (<23 cm), normal (23–24.9 cm) and obese (25 more).

### Data collection procedure

Interviewer-administered pre-tested questionnaire was used to collect the data. MUAC was measured at the time of interviews and maternal medical charts were reviewed to collect information on hemoglobin.

### Data analysis

Data were coded, cleaned and entered into SPSS version 20. Frequencies were run to check for accuracy, outliers, inconsistencies and missed values and variables. Then, the data were exported to STATA version 12 for analysis. Univariate logistic regression analysis was employed for the initial inspection of the associations between the outcome and independent variables. Confidence intervals, at the 95 % level were reported for each unadjusted and adjusted OR.

The analyses were repeated in order to determine association of risk factors in early and late onset of preeclampsia [11]. There was no collinearity between explanatory variables as the Mean Variance Inflation Factor (MVIF) was 1.3 and the variance inflation factor for each individual variable was less than 2.

Multivariate logistic regression analyses were employed to control for confounding and stratified analysis were performed to evaluate effect measure modification by age. In looking at the putative association between MUAC and preeclampsia, maternal age, residence, marital status, occupation and monthly income were anticipated as potential confounders. Hence all the covariates were entered into the multivariate logistic regression model.

Age was found to be an effect modifier; thus it was evaluated through stratification into broad age groups and several stratum specific homogenous categories. The results were compared among different categories of age for three forms of preeclampsia. Finally, a separate age stratified and adjusted analysis of the covariates was conducted and the result of this analysis is discussed in detail.

## Results

### Socio-demographic characteristics of study participants

A total of 453, (151 cases and 302 controls) pregnant women who came for antenatal follow up or skilled delivery services were enrolled in this study. The aggregated mean age of the study participants was 27.14 years (28.14 ± 6.3 for cases and 26.63 ± 5.4 for controls), ranging between 16 and 43 years. Relatively higher proportions of cases than controls were older, uneducated and urban residents (Table 1).

**Table 1:** Socio-demographic characteristics of women attending ANC and/or skilled delivery service in Bahir Dar City, October 2014

Variables	Preeclampsia		#COR (95 % CI)				
	Cases # (%)	Controls # (%)					
			Preeclampsia All cases	Early onset Preeclampsia		Late onset Preeclampsia	
Individual factors							
Age							
< 35 years	123 (82)	268 (88.7)	Base	39	Base	84	Base
> = 35 years	28 (18.0)	34 (11.3)	2.04 (1.02, 4.07)	9	1.18 (0.92, 1.52)	19	1.77 (0.99,3.26)
Residence							
Urban	82 (54.3)	195 (64.6)	Base	26	Base	56	Base
Rural	69 (45.7)	107 (35.4)	1.53 (1.03, 2.28)	22	1.54 (0.83, 2.85)	47	1.56 (0.99,2.45)
Marital status							
Married	141 (93.4)	281 (93.0)	Base	45		96	Base
Single^a^	10 (6.6)	21 (7.0)	0.95 (0.43, 2.06)	3	1.03 (0.42, 2.49)	7	1.00 (0.40,2.35)
Occupation							
Employed	33 (21.8)	72 (23.8)	Base	12		21	Base
Merchant	25 (16.6)	44 (14.6)	1.21 (0.65, 2.35)	8	1.09 (0.41, 2.88)	17	1.33 (0.63,2.78)
House wife	93 (61.6)	186 (61.6)	1.17 (0.54, 2.54)	28	0.90 (0.43, 1.87)	65	1.21 (0.69,2.13)
Education							
Unable to read & write	70 (40.9)	68 (24.1)	2.18 (1.19, 4.62)	27	1.11 (0.70 1.73)	44	1.12 (0.71,1.76)
Able to read & write	101 (59.1)	214 (75.9)	Base	21	Base	59	Base
Monthly Income							
< 1500 Birr	88 (58.3)	170 (56.3)	0.92 (0.62, 1.37)	28	0.92 (0.49, 1.70)	60	1.09 (0.70,1.73)
> 1500 Birr	63 (41.7)	132 (43.7)	Base	20	Base	43	Base

^a^Unmarried, widow and divorced

### Maternal body size and dietary characteristics

Among participants, higher proportion (49 %) of the cases had a higher MUAC (≥25 cm) measure than controls (35 %). Furthermore, higher proportions of cases (82.1 %) were reported to have consumed coffee daily than controls (65.6 %). Significantly more proportions of controls were reported to have taken fruits, vegetables and folates during pregnancy than cases (Table 1). The majority of cases (96 %) and controls (95.4 %) had ANC attendance during their last pregnancy.

### The effect of maternal socio-demographic characteristics, body size and dietary habits on preeclampsia

The Univariate analysis in this study revealed several factors that are associated with overall preeclampsia. Illiteracy (COR = 2.18, 95 % CI: 1.19, 4.62), rural residence (COR = 1.53, 95 % CI: 1.03, 2.28), anemia (COR = 3.96, 95 % CI: 1.12, 2.46), intake of vegetables (COR = 0.31, 95 % CI: 0.12, 0.50) and/or meat/animal product (COR = 1.34, 95 % CI: 1.06, 2.28) were the covariates that were significantly associated with overall preeclampsia in the Univariate model (Tables 1 and 2). However, these factors turned out to be insignificant in the multivariate logistic regression model (Table 3).

**Table 2:** Univariate analysis of nutritional, dietary and lifestyle factors with incidence of preeclampsia, Bahir Dar City, October, 2014

Variables	Preeclampsia		#COR (95 % CI)				
	Yes # (%)	No # (%)	Overall preeclampsia	Early Pre-eclampsia		Late onset Preeclampsia	
Nutritional, dietary and lifestyle factors							
MUAC							
< 23 cm	41 (27.2)	115 (38.0)	Base	16	Base	25	Base
23–24.9 cm	36 (23.8)	82 (27.2)	1.23 (0.72,2.09)	13	1.14 (0.52, 2.49)	23	1.27 (0.67, 2.39)
25 cm and above	74 (49.0)	105 (34.8)	1.98 (1.24, 3.14)	19	1.30 (0.63, 2.66)	55	2.67 (1.38, 4.07)
Fruit intake							
Yes	92 (60.9)	247 (81.8)	0.35 (0.22,0.54)	21	0.29 (0.15, 0 .54)	38	0.37 (0.22, 0.60)
No	59 (39.1)	55 (18.2)	Base	27	Base	65	Base
Vegetable intake							
Yes	105 (69.5)	266 (88.0)	0.31 (0.12, 0.50)	31	0.35 (0.19, 0.60)	74	0.35 (0.20, 0.61)
No	46 (30.5)	36 (12.0)	Base	17	Base	29	Base
Meat/animal product intake							
Yes	143 (94.7)	281 (93.0)	1.34 (1.06, 2.28)	44	1.22 (0.39, 3.71)	99	1.86 (0.62, 5.56)
No	8 (5.3)	21 (7.0)	Base	4	Base	4	Base
Coffee intake							
Yes	124 (82.1)	198 (65.6)	2.41 (1.49,3.90)	43	4.51 (1.73, 11.75)	81	1.97 (1.12, 3.22)
No	27 (17.9)	104 (34.4)	Base	5	Base	22	Base
Folate intake							
Yes	99 (65.6)	283 (93.7)	0.13 (0.07,0.23)	31	0.12 (0.06, 0.26)	68	0.13 (0.07, 0.24)
No	52 (34.4)	19 (6.3)	Base	17	Base	35	Base
Anemia (*n* = 323)							
Yes	18 (14.9)	10 (4.9)	3.96 (1.54, 10.17)	5	1.11 (0.13, 9.27)	13	5.52 (2.11, 14.44)
No	103 (85.1)	192 (95.1)	Base	47	Base	38	
Alcohol intake							
Yes	80 (53.0)	122 (40.4)	1.66 (1.12, 2.46)	26	1.63 (1.04, 2.55)	54	1.61 (1.03, 2.52)
No	71 (47.0)	180 (59.6)	Base	22	Base	49	Base
Physical exercise							
Yes	118 (78.1)	254 (84.1)	0.68 (0.42, 1.11)	40	0.59 (0.32, 1.12)	78	0.64 (0.34, 1.03)
No	33 (21.9)	48 (15.9)	Base	8	Base	25	Base

**Table 3:** Result of multivariate logistic regression analysis of covariates with early onset, late onset and overall incidence of preeclampsia, Bahir Dar City, October, 2014

Covariates	All Preeclampsia	Early Onset Preeclampsia	Late Onset Preeclampsia
	(*n* = 151)	(*n* = 48)	(*n* = 103)
	Adjusted OR (95 % CI),	Adjusted OR (95 % CI),	Adjusted OR (95 % CI),
Age			
< 35 years	Base^a^	Base	Base
> = 35 years	2.07 (1.02, 4.19)	1.27 (0.48, 3.38)	1.56 (0.74, 3.24)
Marital status			
Married	Base		Base
Rural	1.43 (0.78, 2.68)	1.27 (0.49, 3.28)	1.13 (0.60,2.11)
MUAC			
< 23 cm	Base	Base	Base
23–24.9 cm	1.63 (0.84,2.85)	1.37 (0.54, 3.48)	1.24 (0.69,2.56)
25 cm and above	3.33 (1.87, 5.79)*	1.98 (0.79, 4.94)	3.63 (1.89,6.97)*
Fruit intake			
Yes	0.47 (0.27,0.83)*	0.32 (0.11, 0.96)	0.34 (0.16, 0.71)*
No	Base	Base	Base
Vegetable intake			
Yes	0.59 (0.30, 1.15)	0.48 (0.17, 1.35)	0.21 (0.90, 3.71)
No	Base	Base	Base
Coffee intake			
Yes	2.14 (1.32, 3.46)*	2.36 (0.84, 6.62)	1.53 (0.84, 2.83)
No	Base	Base	Base
Folate intake			
Yes	0.18 (0.10, 0.32)*	0.12 (0.04, 0.33)*	0.16 (0.08, 0.33)*
No	Base	Base	Base
Anemia (*n* = 323)			
Yes	1.89 (0.64, 5.61)	1.19 (0.11, 12.82)	2.97 (0.91, 9.69)
No	Base	Base	Base
Alcohol intake			
Yes	1.31 (0.79, 2.18)	1.21 (0.56, 2.62)	0.69 (0.39, 1.20)
No	Base	Base	Base
Meat/animal product intake			
Yes	1.53 (0.56,4.20)	1.00 (0.25, 4.07)	3.07 (0.51, 18..66)
No	Base	Base	Base
Physical exercise			
Yes	0.28 (0.02, 2.99)	0.85 (0.32, 2.26)	0.69 (0.32, 7.00)
No	Base	Base	Base

Adjusted for maternal age, * statistically significant at *P* value <0.05 with 95 % CI

^a^Reference category

Similar results were observed when socio-demographic characteristics, body size and dietary habits were analyzed with late onset preeclampsia. Advanced maternal age (COR = 1.77, 95 % CI: 1.00, 3.26), rural residence (COR = 1.56, 95 % CI: 1.00, 2.45), vegetable consumption (COR = 0.35, 95 % CI: 0.20, 0.61) and daily consumption of coffee (COR = 1.97, 95 % CI: 1.12, 3.22) were the covariates significantly associated with late onset preeclampsia in the Univariate model; however, none of these covariate retain their significance in the adjusted model (Table 2). On the other hand, vegetable intake (COR = 0.35, 95 % CI: 0.19, 0.60) and alcohol consumption (COR = 1.63, 95 % CI: 1.04, 2.55) were also associated with early onset preeclampsia.

Since the association of preeclampsia with dietary pattern may be related with other covariates, variables which had statistical significance in the Univariate logistic regression model were entered into the multivariate logistic regression model separately for overall and late onset preeclampsia.

After controlling for confounding factors, including residence, education, anemia, alcohol intake and consumption of meat, five covariates remain significantly associated with overall preeclampsia.

The likelihood of overall preeclampsia was evident for women who had a higher MUAC measure. Obese women (MUAC ≥ 25 cm) were three times more likely than their normal (MUAC 23–24.99 cm) and underweight (MUAC < 23 cm) counterparts to have overall preeclampsia (AOR = 3.33, 95 % CI: 1.87, 5.79). The odds of overall preeclampsia were higher among advanced age women (AOR = 2.07, 95 % CI: 1.02, 4.19). Women who reported to have taken coffee daily were two times more likely to develop preeclampsia than who did not (AOR = 2.14, 95 % CI:1.32, 3.46). The likelihood of overall preeclampsia was prevented by 53 and 82 % among women reported to have compliant to folate supplement (AOR = 0.47, 95 % CI: 0.27, 0.83) and among women reported to have adequate fruit intake prior to or during pregnancy (AOR = 0.18, 95 % CI: 0.10, 0.32) (Table 3).

Except for advanced maternal age and coffee consumption, obesity (AOR = 3.63, 95 % CI: 1.89, 6.97), fruit intake (AOR = 0.34, 95 % CI: 0.16, 0.71) and compliance to folate supplement (AOR = 0.16, 95 % CI: 0.08, 0.33) have shown odds of similar magnitude with late onset preeclampsia in the multiple logistic regression model.

However, obesity did not show any association with early onset preeclampsia (AOR = 1.30, 95 % CI: 0.63, 2.66) while fruit consumption and compliance to folate supplement have shown a protective effect against early onset preeclampsia (AOR = 0.32, 95 % CI: 0.11, 0.96) and (AOR = 0.12, 95 % CI: 0.04, 0.33), respectively (Table 3).

Factors such as marital status, monthly income, occupation and physical exercise did not show any significant association with preeclampsia in this study.

### Age stratified analysis of the effect of MUAC on preeclampsia

Based on the stratified analysis, a confounding effect between MUAC and suspected confounders was not observed. However, age was found to have effect modification with MUAC in its relationship with the overall and late onset preeclampsia. Substantial variation was observed between the stratum specific estimates (ORs) of MUAC when stratified by age. The effect of obesity on overall preeclampsia was significant among women younger than 35 years (COR = 1.81, 95 % CI: 1.11, 2.99) (Table 4).

**Table 4:** Result of age stratified crude analysis of the effect of MUAC on preeclampsia, Bahir Dar City, October, 2014

Age	COR	[95 % Conf. Interval]
The effect of MUAC on Overall preeclampsia when stratified by AGE		
< 35 years	1.81	1.11, 2.99^a^
≥ 35 years	0.77	0.21, 2.82
The effect of MUAC on Early onset preeclampsia when stratified by AGE		
< 35 years	1.33	0.62, 2.94
≥ 35 years	0.62	0.10, 4.72
The effect of MUAC on Late onset preeclampsia when stratified by AGE		
< 35 years	2.09	1.16, 3.86^a^
≥ 35 years	0.86	0.20, 4.03

^a^statistically significant at 95 % CI

When age groups were made more homogenous through adjusted stratification, it became evident that obesity was strongly associated with overall preeclampsia among women age ≤24 years (AOR = 2.31, 95 % CI: 1.07, 5.12) and 25–29 years (AOR = 3.16, 95 % CI: 1.21, 8.24). Odds of similar magnitude were observed in the relationship between obesity and late onset preeclampsia in women age ≤24 years and 25–29 years (AOR = 3.66, 95 % CI: 1.37, 10.87) and (AOR = 1.98, 95 % CI: 1.16, 3.40), respectively (Table 5).

**Table 5:** Result of age stratified and adjusted analysis of covariates significantly associated with overall, early onset and late onset preeclampsia, Bahir Dar City, October, 2014

Covariates	Overall Preeclampsia		Early onset Preeclampsia		Late onset preeclampsia	
	OR	95 % CI	OR	95 % CI	OR	95 % CI
	Age ≤ 24 years					
MUAC: Normal vs underweight	1.07	0.17, 6.67	0.30	0.07, 1.34	1.53	0.71, 3.29
Obese vs underweight	2.31,	1.06, 5.12^a^	1.00	0.66, 23.93	3.66	1.37, 10.87^a^
Fruit intake: yes vs no	0.30	0.13, 0.72^a^	0.71	0.17, 3.18	0.25	0.09, 0.68^a^
Adherence to folate: yes vs no	0.16	0.05, 0.46^a^	0.71	0.17, 3.18	0.09	0.02, 0.42^a^
Coffee consumption : yes vs no	4.39	1.32, 14.56^a^	0.67	0.13, 3.34	3.87	1.31, 13.79^a^
	Age 25–29 years					
MUAC: Normal vs underweight	2.21	0.58, 8.47	5.75	0.64, 51.57	1.89	0.68, 5.26
Obese vs underweight	3.16	1.21, 8.24^a^	5.16	0.55, 48.40	1.98	1.16, 3.40^a^
Fruit intake: yes vs no	0.74	0.26, 2.12	0.52	0.12, 2.33	0.64	0.22, 1.81
Adherence to folate: yes vs no	0.11	0.03, 0.38^a^	0.86	0.08, 8.54	0.09	0.03, 0.30^a^
Coffee consumption : yes vs no	1.24	0.51, 3.01	0.25	0.03, 2.09	–	–
	Age 30–34 years					
MUAC: Normal vs underweight	1.16	0.40, 3.35	2.76	0.20, 36.77	0.64	0.42, 3.94
Obese vs underweight	1.04	0.26, 4.13	2.86	0.26, 31.53	0.91	0.42, 1.97
Fruit intake: yes vs no	0.41	0.11, 1.49	0.04	0.03, 1.49	0.70	0.16, 2.97
Adherence to folate: yes vs no	0.12	0.03, 0.47^a^	0.03	0.02, 0.27^a^	0.20	0.04, 0.93^a^
Coffee consumption : yes vs no	1.14	0.29, 4.51	0.50	0.04, 6.63	–	–
	Age >35 years					
MUAC: Normal vs underweight	0.38	0.06, 2.38	0.37	0.03, 4.06	0.90	0.16, 5.04
Obese vs underweight	3.45	0.53, 22.54	2.87	0.21, 39.07	1.57	0.62, 3.97
Fruit intake: yes vs no	0.21	0.05, 0.94^a^	0.15	0.01, 1.73	0.22	0.05, 1.05
Adherence to folate: yes vs no	0.23	0.03, 1.54	0.21	0.02, 1.87	0.13	0.02, 0.80^a^
Coffee consumption : yes vs no	3.67	0.52, 25.69	Omitted		–	–

^a^Statistically significant after controlling for age through adjusted stratification

With regard to age stratified adjusted relationship between folate intake and preeclampsia, fruit intake and compliance to folate supplement during pregnancy have shown a consistent protective effect against overall and late onset preeclampsia across almost all age groups. The odds of developing preeclampsia was 70 and 75 % lower among women who used fruit in their diet as compared to non-users for overall and late onset preeclampsia respectively.

The odds of developing overall and late onset preeclampsia was 70 and 75 % lower among women who used fruits in their diet as compared to non-users.

The age stratified adjusted logistic regression analysis shows a significant predictive association between coffee consumption and overall (AOR = 4.39, 95 % CI: 1.32, 14.56) and late onset preeclampsia (AOR = 3.87, 95 % CI: 1.31, 13.79) among young (≤24 years) women (Table 5). However, except compliance to folate supplement among women age 30–34 years, none of the other covariate were found to be significantly associated with early onset preeclampsia in the age adjusted stratified analysis (Table 5).

## Discussion

This case control study aimed at determining the effect of obesity and dietary habits on preeclampsia among women in Ethiopia. We assessed the effect of a wide range of factors on preeclampsia and found that obesity, advanced maternal age and coffee consumptions are risk factors for preeclampsia. On the other hand, fruit and/or vegetable consumption and compliance to folate supplement were protective factors against preeclampsia.

The study revealed a positive relationship in the risk of pre-eclampsia with increasing mid-arm circumference. Obese (MUAC ≥ 25 cm) women were three times more likely to develop preeclampsia than women in the normal (23–24.99 cm) or underweight (<23 cm) range. The result was consistent with studies that showed obese women are two to three times more likely to develop preeclampsia than their leaner counterparts [11, 12].

The authors checked if the observed relationship between MUAC and preeclampsia was confounded by maternal age or not. The age stratified analysis revealed an effect modification/interaction between MUAC and maternal age. The effect of obesity on overall and late onset preeclampsia was evident only for younger (<35 years) women, mainly for age ≤ 24 years and 25–29 years. This study discovered that obesity has no association with pre-eclampsia among older women as opposed to what is commonly known. When age increased, body size and risk of preeclampsia also increased as supported by other studies [6, 11, 27]. We speculated that an increase in obesity trend among young women might be a strong risk factor underlying the observed incidence of preeclampsia [28]. The rapid shift in population towards a more obese phenotype in recent years might lead to a marked increase in the incidence of overweight and obese women of childbearing age, including preeclampsia in early ages [12]. The possible variation in the distribution of age between cases and controls and the time at which MUAC was measured might attribute to the attenuation of our results. The use of MUAC is also not a sensitive measure of body size [25, 29]. Other than chance, our findings may have at least two possible interpretations: obesity is a risk factor for preeclampsia in young age, or our methodological limitations might have created a spurious predictive association. However, this study did not find a causal relationship between obesity and early onset preeclampsia. Large-scale population based studies using BMI instead of MUAC may provide evidence to corroborate or refute the current findings. Measures of body composition, including percent body fat mass, may very likely identify obese women at risk of preeclampsia more accurately [12, 29]. However, our findings are useful in predicting risk of obesity for preeclampsia among young women in the country.

Consistent with other studies [30], this study revealed that daily coffee intake during pregnancy has statistically significant association with the occurrence of overall preeclampsia. Studies in Norway [15, 19] found higher caffeine (coffee or tea) intake during pregnancy is associated with elevated systolic blood pressure levels in first and third trimester pregnancy. The mechanism by which caffeine exposure affects heart rate and blood pressure levels might include increase of catecholamine levels, which may subsequently lead to vasoconstriction. Differences in blood pressure levels associated with caffeine intake during pregnancy might be markers of subclinical cardiovascular adaptation mechanisms and the subsequent risk of hypertensive complications, such as pregnancy-induced hypertension and pre-eclampsia [18, 31]. Some studies reported that caffeine intake only increases incidence of systolic hypertension during the first trimester pregnancy, but did not have any effect later or on diastolic blood pressure [32]. However, other studies did not find any evidence on the effects of caffeine intake on blood pressure during pregnancy [31]. In this study, the effect of coffee consumption was apparent only for late onset and overall preeclampsia among younger (≤24 years) women. This observation might be due to the sample size and lack of homogeneity of the data distribution between cases and controls. Employing a robust longitudinal design by recruiting a larger sample size may provide credible insight in this regard. Analyzing the amount and frequency of daily caffeine intake, including Coca-Cola and tea may provide more insightful results.

Maternal diet is one of the factors found to play a role in the etiology of pre-eclampsia. In a previous study [19], it was found that high scores on a healthy diet characterized by high intake of vegetables, fruits and vegetable oils were associated with reduced risk of pre-eclampsia in nulliparous women. The findings of this study also revealed that women who eat fruit had a lower risk of developing early onset, late onset and overall preeclampsia than women who did not. This finding is consistent with studies conducted in Norway [8, 13, 15, 18, 19] which reported a reduced risk of preeclampsia with organic vegetable consumption. There are several mechanisms for a biological effect of dietary behavior on the incidence of preeclampsia. Fruits are rich in micronutrients such as antioxidants, vitamins, minerals, and dietary fibers. A diet rich in fruits and uncooked vegetables decreased the risk of hyperhomocysteinemia, which is one of the risk factors for the occurrence of preeclampsia [33]. However, the protective effect of fruit consumption in the adjusted stratified analysis was apparent only for some age groups (age <20 years and ≥35 years). The mechanism of why fruit consumption has an effect on preeclampsia primarily among very young and older women is not well understood and needs further study. This finding might be due to the variation in the distribution of samples and other unforeseen methodological issues. Intake of vegetable also has a non-significant but an important protective effect against all forms of preeclampsia. In contrast, other previous studies did not find supporting evidence that self-reported intake of organic fruits and vegetables prevent preeclampsia [20, 34]. Therefore, the researchers in this study believe that the effect of dietary components, including macronutrients, micronutrients, dietary fiber and individual foods as well as overall food patterns and qualities must be assessed using large-scale longitudinal observational studies.

Consistent with previous studies [35, 36], we found that compliance to folate supplementation during pregnancy was associated with a reduced risk of all forms preeclampsia. It was interesting that compliance to folate supplement has shown a protective effect against all forms of preeclampsia across all age categories. Folate supplementation during pregnancy decreases plasma homocysteine concentrations and improves endothelial function in adults with hyperhomocysteinemia [36, 37]. Homocysteine is an amino acid released as the body digests dietary protein. It has been shown that its level increases during preeclampsia and elderly. Too much homocysteine in the blood is linked with a greater risk of heart disease, stroke and nerve damage [8]. Excessive homocysteine in pregnancy might damage the vascular endothelium of the developing placenta by promoting oxidative stress, thereby increasing contractile response and the production of procoagulants and vasoconstrictors which lead to the development of preeclampsia [33]. These findings underscore the need for continued promotion of universal provision of folate for all pregnant women. However, research findings on the effect of folate during pregnancy are controversial. Some studies revealed that taking folate would lead to childhood asthma [38] and reduced fetal growth [39], whereas others did not find any relationship [20]. A prospective observational cohort study may provide more robust evidence.

Unlike other studies [6], maternal education, occupation and residence did not show a significant association with preeclampsia in the current study. Such difference could be due to the variation in study design, sample size and study settings. The effect of anemia and intake of meat and animal product did not turn out significant in the final models either. Residual confounding due to insufficient information or unmeasured lifestyle habits might be involved. Therefore, further research using large sample and validated questionnaires is needed.

Numerous researches have shown that there is no one best way to prevent preeclampsia, though for many years body size and diet have been claimed to play a role in preeclampsia [12, 15]. Increase in the amount of fruits and vegetables and decreasing the amount of fried foods and junk foods, getting enough rest, exercising regularly, avoiding alcohol, avoiding beverages containing caffeine, and taking folate and iron regularly has been recommended by various studies [12, 18, 19, 34, 40]. The result of this study also supports the above recommendations, including the need to emphasize on prevention of obesity in young women.

The findings of this study should be viewed in light of the following limitations: the use of survivor sampling method to recruit controls might introduce selection bias which leads to heterogeneous distribution of some covariates between cases and controls. As a result, it was impossible to make a comparison between early and late onset preeclampsia. Measurement of MUAC was made at the time of data collection and the effect of pregnancy on body size was not considered. Therefore, the MUAC measurement may not necessarily indicate the pre-conception value. In case-control studies, recall bias and lack of temporal relationship are inherent limitations. The relatively small number of our sample might dilute the potential true effect of some covariates. Hence, generalization of the findings in this study may not be possible.

## Conclusions

Obesity in young age is a risk factor for late onset and overall preeclampsia while compliance to folate supplement and adequate fruit consumption are protective factors. The associated risk and protective factors identified in this study are modifiable. Lifestyle modifications, including weight reduction, before, during and after pregnancy are recommended to reduce the risk of preeclampsia among young women. Universal provision and compliance to folate supplement and fruit consumption should always be promoted for pregnant women. Robust research with larger sample size and well-designed methodology needs to be conducted to corroborate our findings.
